# Presence of three dengue serotypes in Ouagadougou (Burkina Faso): research and public health implications

**DOI:** 10.1186/s40249-016-0120-2

**Published:** 2016-04-05

**Authors:** Valéry Ridde, Isabelle Agier, Emmanuel Bonnet, Mabel Carabali, Kounbobr Roch Dabiré, Florence Fournet, Antarou Ly, Ivlabèhiré Bertrand Meda, Beatriz Parra

**Affiliations:** Department of Social and Preventive Medicine, University of Montreal School of Public Health (ESPUM), Montréal, Canada; University of Montreal Public Health Research Institute (IRSPUM), Pavillon 7101 Avenue du Parc, P.O. Box 6128, Centre-ville Station, Montreal, Quebec H3C 3J7 Canada; Identités et Différenciations de l’Environnement des Espaces et des Sociétés – Caen (IDEES), University of Caen Basse-Normandie, Caen, France; International Vaccine Institute, Dengue Vaccine Initiative, SNU Research Park, 1 Gwanak-ro, Gwanak-gu, Seoul, 151-742 Korea; Institut de Recherche en Sciences de la Santé (IRSS), B.P. 545 Bobo-Dioulasso, Burkina Faso; Unité Maladies infectieuses et vecteurs : écologie, génétique, évolution et contrôle (MIVEGEC), Institut de recherche pour le développement (IRD), B.P. 171 Bobo-Dioulasso, Burkina Faso; Institut de Recherche en Sciences de la Santé (IRSS), 03 B.P. 7192 Ouagadougou, Burkina Faso; Grupo de Virus Emergentes y Enfermedad, Departamento de Microbiología Universidad del Valle, Cali, Colombia

**Keywords:** Dengue, Fever, Acute febrile non-malaria cases, Burkina Faso, *Aedes*, Health system, Cost, Mobility

## Abstract

**Background:**

The significant malaria burden in Africa has often eclipsed other febrile illnesses. Burkina Faso’s first dengue epidemic occurred in 1925 and the most recent in 2013. Yet there is still very little known about dengue prevalence, its vector proliferation, and its poverty and equity impacts.

**Methods:**

An exploratory cross-sectional survey was performed from December 2013 to January 2014. Six primary healthcare centers in Ouagadougou were selected based on previously reported presence of Flavivirus. All patients consulting with fever or having had fever within the previous week and with a negative rapid diagnostic test (RDT) for malaria were invited to participate. Sociodemographic data, healthcare use and expenses, mobility, health-related status, and vector control practices were captured using a questionnaire. Blood samples of every eligible subject were obtained through finger pricks during the survey for dengue RDT using SD BIOLINE Dengue Duo (NS1Ag and IgG/IgM)® and to obtain blood spots for reverse transcription polymerase chain reaction (RT-PCR) analysis. In a sample of randomly selected yards and those of patients, potential *Aedes* breeding sites were found and described. Larvae were collected and brought to the laboratory to monitor the emergence of adults and identify the species.

**Results:**

Of the 379 subjects, 8.7 % (33/379) had positive RDTs for dengue. Following the 2009 WHO classification, 38.3 % (145/379) had presumptive, probable, or confirmed dengue, based on either clinical symptoms or laboratory testing. Of 60 samples tested by RT-PCR (33 from the positive tests and 27 from the subsample of negatives), 15 were positive. The serotypes observed were DENV2, DENV3, and DENV4. Odds of dengue infection in 15-to-20-year-olds and persons over 50 years were 4.0 (CI 95 %: 1.0–15.6) and 7.7 (CI 95 %: 1.6–37.1) times higher, respectively, than in children under five. Average total spending for a dengue episode was 13 771 FCFA [1 300–67 300 FCFA] (1$US = 478 FCFA). On average, 2.6 breeding sites were found per yard. Potential *Aedes* breeding sites were found near 71.4 % (21/28) of patients, but no adult *Aedes* were found. The most frequently identified potential breeding sites were water storage containers (45.2 %). Most specimens collected in yards were *Culex* (97.9 %).

**Conclusions:**

The scientific community, public health authorities, and health workers should consider dengue as a possible cause of febrile illness in Burkina Faso.

**Electronic supplementary material:**

The online version of this article (doi:10.1186/s40249-016-0120-2) contains supplementary material, which is available to authorized users.

## Multilingual abstracts

Please see Additional file 1 for translations of the abstract into the six official working languages of the United Nations.

## Background

West African countries are carrying an enormous malaria burden [1]. In Burkina Faso alone, the number of malaria deaths is estimated at nearly 40 000 annually [2]. Several interventions have been put in place to reduce that burden, and the results thus far have been positive, with malaria on the decline since 2004 [2, 3]. Malaria control is likely to improve exponentially over the coming years due to three key interventions: 1) mass distributions of long-lasting insecticidal nets (LLINs) in 2010 and 2013 [4]; 2) the use of malaria rapid diagnostic testing (RDT) in all public healthcare centers since 2012; and 3) malaria treatment in the form of Artemisinin-based combination therapy (ACT) administered routinely by community health workers (CHWs) since 2010 [5]. As such, the proportion of febrile under-five children given antimalarials rose from 35 % in 2010 to 49.2 % in 2014, and even up to 66.1 % in certain regions [6–8].

The undeniable magnitude of the malaria burden undoubtedly contributes to the lesser concern shown by decision-makers, health workers, and researchers for other febrile illnesses [9]. Some have stressed the need for “deconstructing ‘malaria’ in West Africa” [10]. Health workers are still trained with the idea that all fever is synonymous with malaria. Yet fever, a major driver for healthcare center consultations, can be symptomatic of several illnesses, including not only malaria, but also diarrhea, typhoid, or even dengue [11, 12]. Indeed, the World Health Organization (WHO) worries that “*dengue continues to be underreported in Africa owing to a lack of awareness among health-care providers, the presence of other febrile illnesses (especially malaria)…*” [13]. In Africa, there is growing interest in fever not associated with malaria, as clearly shown in recent studies in Tanzania and Senegal [12, 14]. However, dengue is a febrile illness that resembles several others, including malaria [15, 16]. Even though the global impact of dengue is immense, there is still very little known about its prevalence and burden in Africa [15, 17, 18].

The first dengue epidemic in Burkina Faso occurred in 1925 [17]. Later, a significant number of cases were seen in the 1980s [17, 19] and identified as DENV2 [19, 20]. In the 2000s, DENV1 was found among travellers returning from Burkina Faso [21]. In fact, Burkina Faso is one of the 34 African countries in which dengue cases have been reported since the 2000s [15, 17, 22]. A 2003 study of 191 blood donors and 492 pregnant women in two districts, one rural (Nouna) and one urban (Ouagadougou), showed that between 26 and 39 % of those surveyed had been in contact with the dengue virus [23]. Another study, conducted in 2004 with 3 000 children in Ouagadougou, found that 22 % of them had been in contact with a virus of the Flavivirus family, to which the dengue virus belongs [24]. More recently, an epidemic broke out in 2013, especially in the capital [25, 26]. Moreover, DENV3 was identified in a European patient who had travelled in Burkina Faso in 2013 [27] and in a sample of 43 patients of two health facilities in the capital in 2013 [25].

In Burkina Faso, *Aedes aegypti* is the primary known dengue vector in urban areas [20]. However, whereas knowledge about vector species is essential for the development of strategies to control a disease like dengue, the fact is that our knowledge is old and not very up-to-date. Yet rampant population growth, poorly planned urbanization, and the circulation of people and goods are all factors that can encourage its emergence and the arrival of new vectors such as *Aedes albopictus*, which is known to be very invasive, based on what is currently being observed in central Africa [28, 29].

So, we have only very limited knowledge at this time about the dengue virus in Africa in general [11] and in Burkina Faso in particular [9]. Before 2013, dengue was not taken into account in health statistics and was not among the diseases requiring notification in the surveillance system [30]. Then, in 2013, the African Union called for implementation of dengue control interventions. However, more effective development of dengue control strategies requires—beyond international best practices [31] and WHO recommendations [32]— local evidence to support the country’s decision-makers. We therefore conducted a cross-sectional study in the capital to gain a better understanding of the epidemiological, clinical, entomological, and public health situations with regard to dengue and its vector in acute febrile non-malaria cases.

## Methods

### Design and population

**Fig. 1:**
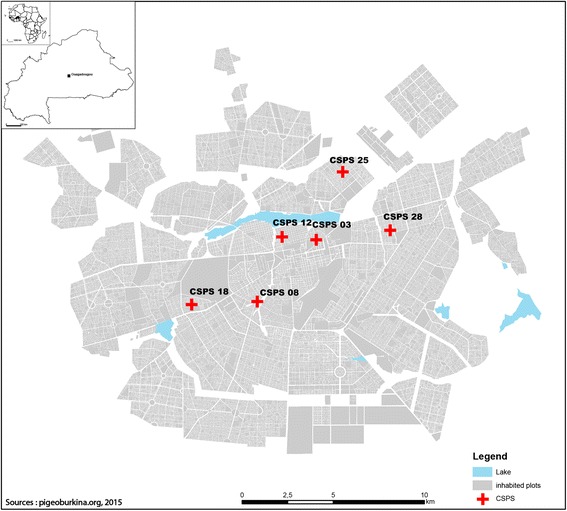
Study map

An exploratory cross-sectional survey was conducted between December 9, 2013, and January 4, 2014. Five sectors and six corresponding primary healthcare centers (CSPSs) in Ouagadougou, the capital of Burkina Faso (Fig. 1), were selected based on previously reported presence of Flavivirus [24]: CSPS 3 and 12 (Dapoya), 8 (Gounghin), 18 (Pissy), 25 (Somgande), and 28 (Dassasgho).

### Selection criteria

All patients consulting with fever (axillary temperature ≥ 38 °C) at the moment of the survey or with history of fever within the previous week and with a negative rapid diagnostic test (RDT) for malaria were invited to participate in the study.

### Data and sample collection

Sociodemographic data, presence and history of fever, healthcare use and expenses, mobility (local and international), health-related status (including current symptoms), and information about vector control practices were obtained through the administration of a structured questionnaire by trained nurses. Blood samples of every eligible subject (*n* = 379) were obtained through finger pricks during the survey to perform a dengue rapid diagnostic test (NS1Ag and IgG/IgM) and to obtain blood spots for RT-PCR analysis.

### Rapid diagnostic testing

Following aseptic preparation of the subject, finger prick blood samples were obtained and introduced into each of the two cassettes of the commercially available kit SD BIOLINE Dengue Duo (Standard Diagnostics, Seoul, South Korea)® followed by the addition of the assay diluents. Results were read 15–20 min after performing each test; in cases of invalid results, another procedure was conducted with a new cassette. All procedures were conducted according to the manufacturer’s indications.

### RT-PCR analysis

Finger prick blood samples were collected in filter paper (Whatman® 3MM) from every subject with a positive dengue RDT result. Additionally, a sample was collected from every tenth subject with a negative result to see whether it was possible to identify virus presence in patients with negative RDT (i.e., false negative). These filter papers, when dried, were stored individually in a Ziploc® bag in a dry cool place between 4 and 15 °C and subsequently used for RT-PCR analysis at the microbiology laboratory at the Universidad del Valle (Cali, Colombia). The DENV RNA was detected by a conventional DENV-1–4 nested RT-PCR protocol. The viral RNA was extracted from the filter-paper eluted blood samples with the QIAamp® Viral RNA kit (QIAGEN, Germantown, MD). The cDNA was prepared by reverse transcription of RNA using reverse transcriptase avian myeloblastosis virus (Promega, Madison, WI) and an antisense primer, followed by two rounds of nested-PCR. The final PCR products were compared with the DNA band size of the assay positive controls (CDC Reference DENV-1–4 strains) [33]. To confirm the dengue specificity of the PCR products amplified from the samples, the PCR amplicons of the correct size were further sequenced by primer extension using the BigDye Terminator v3.1 Cycle Sequencing protocol (Macrogen Inc., South Korea). Nucleotide sequences of the PCR products amplified by RT-PCR corresponded to cDNA sequences of dengue virus serotypes 2, 3 and 4.

### Data analysis

We used the 2009 WHO dengue classification to identify dengue cases [34]. Based on the presence or absence of diagnostic confirmation, we identified: 1) presumptive cases (clinical symptoms without laboratory investigation); 2) probable cases (positive IgM and/or IgG); and 3) confirmed cases (positive AgNS1 and/or RT-PCR). In each of these categories, we identified three groups of increasing severity: dengue without warning signs, dengue with warning signs, and severe dengue.

Some of the symptoms in the WHO classification were either missing or poorly described in the consultation registers, and so we limited our classification to the following symptoms: nausea/vomiting, pain (headache, muscle pain, joint pain), rash, tourniquet test, abdominal pain, lethargy/sleepiness, convulsions, and mucous membrane bleeding. In infants, diarrhea and coughing were also included. Among dengue cases without warning signs (presumptive, probable, or confirmed), we also considered co-existing conditions (pregnancy, nursing infant, advanced age, diabetes mellitus, HTA, sickle-cell anemia, and cardiac or renal disease) that placed them at risk.

Patients’ socio-economic characteristics (access to water, waste management, possession of durable goods) were used to construct income quantiles using principal component analysis (PCA). It was performed using a tetrachoric correlations matrix (adapted to category variables), and sampling adequacy was assessed using the overall KMO index (0.79). Income terciles were constructed, as the variability of factorial scores did not allow for isolating quintiles or quartiles. Two binary outcomes were then constructed based on the dengue classification. The first outcome was assigned a value of 0 for cases identified without dengue and 1 otherwise, whereas the second outcome was assigned a value of 1 for probable and confirmed cases of dengue and 0 otherwise. Patients’ sociodemographic and clinical characteristics were compared (bivariate analysis) according to these two classifications using chi-squared testing. The second outcome then underwent multiple logistic regression that included all the variables associated in bivariate relation to it with a *p* <0.25 [35]. Based on the complete model, we successively eliminated non-significant variables using likelihood ratio tests. The significance level was set at 0.05, and all data were analyzed with Stata software, version 13.

### Healthcare utilization and costs

We calculated expenses for transportation, drugs, laboratory tests, as well as total expenses by adding together the expenses for all services used over the course of a single dengue episode. This information was obtained during a follow-up visit to the patient within 30 days following diagnosis.

### Mobility

Patients’ mobility was assessed during the first day of consultation and was analyzed based on their reports of travel undertaken in the 15 days preceding the consultation. These trips were coded to determine their number and duration, and whether they occurred inside or outside of the administrative sector in which the patients resided.

### Entomological survey

Potential *Aedes* breeding sites were sought and characterized in randomly selected yards and in the yards of RDT positive subjects; these yards consisted of the peri-domiciliary area on all sides of each dwelling, generally bounded by a fence, wall, or other enclosure, and also included any area within the dwelling that might be used to store water. Larvae were collected and brought back to the laboratory of the *Institut de Recherche en Sciences de la Santé* (IRSS) in Bobo-Dioulasso to monitor the emergence of adults. Our aim was to identify the species and, in the case of *Aedes aegypti*, to condition them, either to be able to detect the presence of the virus or to perform insecticide sensitivity tests (not presented in this article). As well, adult mosquitoes were collected in the morning between 8:00 a.m. and 10:00 a.m. with an electric vacuum cleaner in 20 randomly selected yards in each neighborhood.

A sample of captured mosquitoes was analyzed for DENV presence. Total RNA extraction was performed using Trizol; a DNA copy was obtained using the enzyme SuperScrip II and the primer D2 with a final RNA concentration of 10 ng/uL per sample. The primer D2 was used for the cDNA because it has a high degree of nucleotide correspondence with the four virus serotypes. The cDNA product was amplified using nested PCR with the D1, D2, TS1, TS2, TS3, and TS4, as described by Lanciotti et al. [33]. These analyses were conducted at the CIDEIM vector control unit (Cali, Colombia).

### Ethical considerations

Informed consent was obtained from each subject. The study protocol was reviewed and approved by the National Health Ethics Committee of Burkina Faso and the Institutional Review Board of the CRCHUM in Montreal, Canada.

## Results

**Fig. 2:**
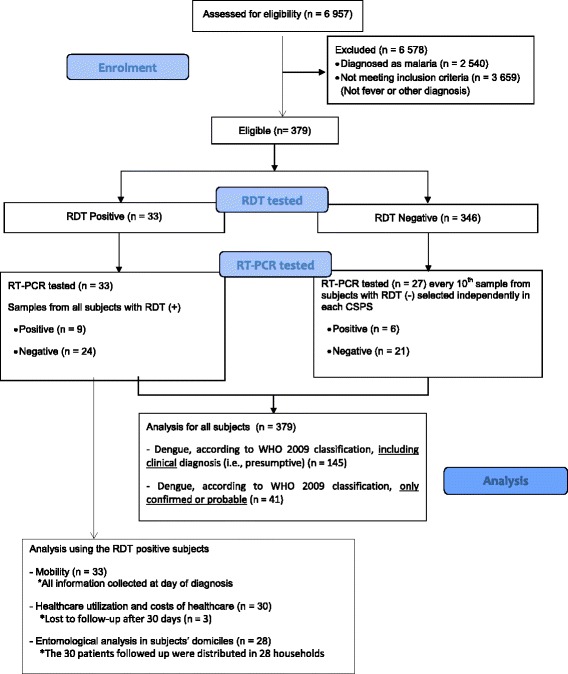
Enrolment and analysis flowchart

From a total of 6 957 people consulting at the study CSPSs, 379 were eligible to participate in the survey (Fig. 2). All patients who satisfied the inclusion criteria consented to take part in the study (379/379). Of these, 59.9 % were women (227/379) and 45.4 % (172/379) were under 15 years of age. The CSPS with the highest rate of enrolment was that of sector 18, with 23.8 % (90/379), followed by that of sector 28, with 21.6 % (82/379) (Table 1). The majority (86.8 %, 329/379) of the patients had a fever of under five days’ duration, and 8.7 % (33/379) had positive RDTs for dengue.

**Table 1:** Sociodemographic and clinical characteristics of patients included in the study

Variables	N	Percentage
Sex (female) (*n* = 379)	227	59.9
Age (range 0–61 years) (*n* = 379)		
• Under 5 years	86	22.7
• 5–14 years	86	22.7
• 15–20 years	31	8.2
• 21–30 years	66	17.4
• 31–40 years	58	15.3
• 41–50 years	34	9.0
• Over 50 years	18	4.7
Healthcare center (*n* = 379)		
• CSPS 3 (Dapoya)	42	11.1
• CSPS 8 (Gounghin)	68	17.9
• CSPS 12 (Dapoya)	26	6.9
• CSPS 18 (Pissy)	90	23.8
• CSPS 25 (Somgande)	71	18.7
• CSPS 28 (Dassasgho)	82	21.6
Income tercile (*n* = 378)		
• Lowest	147	38.9
• Middle	120	31.7
• Highest	111	29.4
Water supply source (*n* = 378)		
• Tap water	278	73.5
• Other	100	26.5
Water storage (*n* = 378)		
• No storage	65	17.2
• Covered containers	296	78.3
• Mixed containers	17	4.5
Waste recuperation service (*n* = 379)		
• Yes	243	64.1
• No	136	35.9
Fever duration^a^ (range 0–37 days) (*n* = 379)		
• Up to 5 days	329	86.8
• More than 5 days	50	13.2
Travel abroad (*n* = 379)		
• No	356	93.9
• Yes	23	6.1

^a^ Self-reported by the patient at time of consultation

### Dengue identification

Table 2 shows that, according to the 2009 WHO classification, 38.3 % (145/379) of the cases had presumptive, probable, or confirmed dengue, based on clinical signs and laboratory results.

**Table 2:** Classification of dengue cases according to WHO 2009 guidelines

Diagnosis according to WHO 2009 classification	Laboratory investigation	N	Proportion (%)
No dengue		234	61.7
Presumptive^a^ dengue without warning signs	–	36	9.5
Presumptive^a^ dengue with warning signs	–	68	17.9
Probable dengue without warning signs	IgG positive	7	1.8
	IgG and IgM positive	11	2.9
Probable dengue with warning signs	IgG positive	1	0.3
	IgG and IgM positive	5	1.3
Confirmed dengue without warning signs	PCR positif	7	1.8
	AgNS1 and PCR positive	3	0.8
Confirmed dengue with warning signs	AgNS1 positive	2	0.6
	PCR positive	3	0.8
	AgNS1 and PCR positive	2	0.6

^a^ According to WHO 2009 guidelines, a presumptive diagnosis is part of the assessment of the case and is only clinically based (i.e., based on signs and symptoms)

Of the 379 patients in the sample, 94 (24.8 %) had a pre-existing medical condition. Among patients classified as dengue cases without warning signs, there was at least one co-occurring condition in 36.1 % (*n* = 13) of the presumptive cases, 38.9 % (*n* = 7) of the probable cases, and 20 % (*n* = 2) of the confirmed cases. Tables 3 and 4 present the different factors associated with dengue infection according to the WHO 2009 guidelines, including clinical/presumptive assessment, as well as probable and confirmed classification of the subjects. In varying the definition of dengue cases (presumptive/probable/confirmed vs. probable/confirmed), only the crude association between age group and dengue was influenced. It became significant (*p* < 0.01) when presumptive cases were excluded from the definition of dengue cases (Tables 3 and 4).

**Table 3:** Sociodemographic and clinical factors associated with dengue infection (dengue vs. no dengue)

Variables	Total (*n* = 379)	Dengue cases^a^ (*n* = 145) (%)	*p*-value
Sex			
• Male	152	58 (38.2)	0.974
• Female	227	87 (38.3)	
Age group			
• Under 5 years	86	31 (36.0)	0.990
• 5–14 years	86	32 (37.2)	
• 15–20 years	31	14 (45.2)	
• 21–30 years	66	26 (39.4)	
• 31–40 years	58	22 (37.9)	
• 41–50 years	34	13 (38.2)	
• 51 years and over	18	7 (38.9)	
Healthcare center			
• CSPS 3 (Dapoya)	42	16 (38.1)	**0.017**
• CSPS 8 (Gounghin)	68	23 (33.8)	
• CSPS 12 (Dapoya)	26	12 (46.2)	
• CSPS 18 (Pissy)	90	22 (24.4)	
• CSPS 25 (Somgande)	71	35 (49.3)	
• CSPS 28 (Dassasgho)	82	37 (45.1)	
Income tercile (*n* = 378)			
• Lowest	147	53 (36.1)	0.762
• Middle	120	46 (38.3)	
• Highest	111	45 (40.5)	
Water supply (*n* = 378)			
• Tap water	278	106 (38.1)	0.982
• Other	100	38 (38.0)	
Waste management			
• Collection service	243	93 (38.3)	0.994
• Other	136	52 (38.2)	
Travel abroad			
• Yes	23	7 (30.4)	0.426
• No	356	138 (38.8)	
Water storage (*n* = 378)			
• No storage	65	24 (36.9)	0.443
• Covered containers	296	111 (37.5)	
• Mixed containers	17	9 (52.9)	
Fever duration			
• Up to 5 days	329	117 (35.6)	**0.006**
• More than 5 days	50	28 (56)	

^a^ Any dengue classification (i.e., presumptive, probable, or confirmed)

Numbers in boldface are *p*-value statistically significant

**Table 4:** Sociodemographic and clinical factors associated with dengue infection (confirmed by RDT, PCR or probable dengue vs. others)

Variables	Total (*n* = 379)	Dengue cases^a^ (*n* = 41)(%)	*p*-value
Sex			
• Male	152	17 (11.2)	0.851
• Female	227	24 (10.6)	
Age group (range 0–61 years)			
• Under 5 years	86	4 (4.7)	**0.001**
• 5–14 years	86	3 (3.5)	
• 15–20 years	31	6 (19.4)	
• 21–30 years	66	8 (12.1)	
• 31–40 years	58	9 (15.5)	
• 41–50 years	34	5 (14.7)	
• 51 years and over	18	6 (33.3)	
Healthcare center			
• CSPS 3 (Dapoya)	42	3 (7.1)	**0.022**
• CSPS 8 (Gounghin)	68	2 (2.9)	
• CSPS 12 (Dapoya)	26	4 (15.4)	
• CSPS 18 (Pissy)	90	6 (6.7)	
• CSPS 25 (Somgande)	71	12 (16.9)	
• CSPS 28 (Dassasgho)	82	14 (17.1)	
Income tercile (*n* = 378)			
• Lowest	147	11 (7.5)	0.195
• Middle	120	14 (11.7)	
• Highest	111	16 (14.4)	
Water supply (*n* = 378)			
• Tap water	278	33 (11.9)	0.286
• Other	100	8 (8.0)	
Waste management			
• Collection service	243	27 (11.1)	0.806
• Other	136	14 (10.3)	
Travel abroad			
• Yes	23	2 (8.7)	0.735
• No	356	39 (11)	
Water storage (*n* = 378)			
• No storage	65	8 (12.3)	0.574
• Covered containers	296	30 (10.1)	
• Mixed containers	17	3 (17.7)	
Fever duration			
• Up to 5 days	329	30 (9.1)	**0.006**
• More than 5 days	50	11 (22)	

^a^ Only cases of probable and confirmed dengue

Numbers in boldface are *p*-value statistically significant

Only healthcare center and age group were significantly associated with dengue infection in multiple logistic regression (table not presented). Thus, compared with the CSPS of sector 8 (Gounghin), the OR for sector 25 (Somgande) was 5.7 (CI 95 %: 1.2–27.4). The ORs of the other CSPSs were not statistically significant. The odds of dengue infection for the 15–20 years and over-50 age groups were 4.0 (CI 95 %: 1.0–15.6) and 7.7 (CI 95 %: 1.6–37.1) times higher, respectively, than for the under-fives.

### RT-PCR serotype identification

**Fig. 3:**
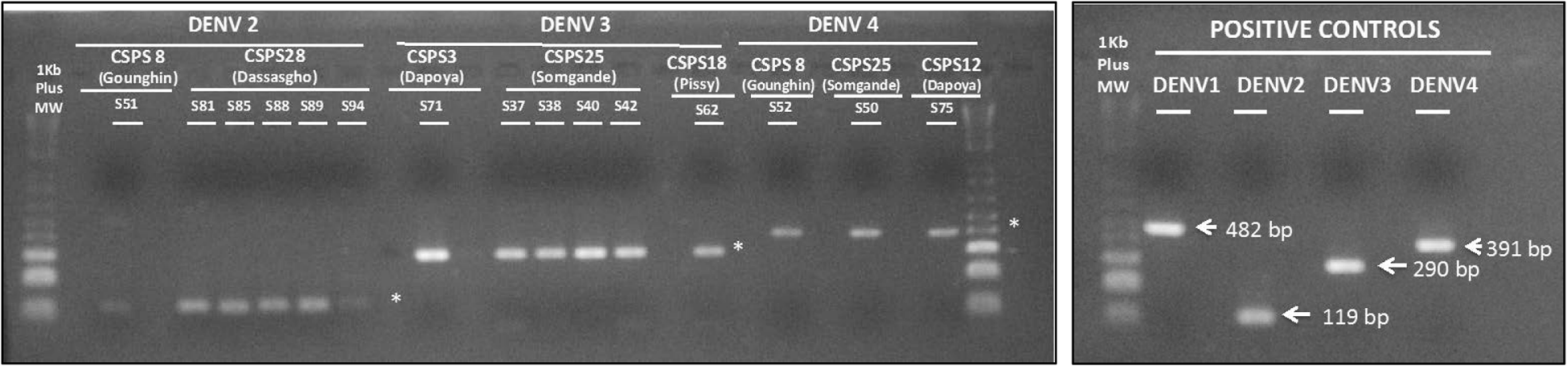
RT-PCR products (2 % Agarose gel)

Of 60 samples tested by RT-PCR (33 from the positive tests and 27 from the subsample of negative tests), 15 were positive: nine from positive RDTs and six from the subsample of negative results (Fig. 3). The serotypes observed, with numbers of cases, were: DENV2 (Dassasgho, *n* = 5; Gounghin, *n* = 1); DENV3 (Dapoya, *n* = 1; Pissy, *n* = 1; Somgande, *n* = 4); and DENV4 (Dapoya, *n* = 1; Gounghin *n* = 1; Somgande, *n* = 1).

Of the 33 subjects with positive RDT results, three were lost to follow-up; consequently, the analyses of healthcare use, costs, and entomology, which involved household visits, were conducted on the remaining 30 subjects.

### Healthcare utilization and costs

Table 5 presents the healthcare resources used by subjects with a positive dengue RDT (*n* = 30) who were followed up within the 30-day period.

**Table 5:** Patients with positive dengue RDT followed up 30 days after diagnosis, by characteristics and service use

Variables	N	Percentage(%)
Diagnosis communicated to the patient (*n* = 30)		
• Dengue	29	96.7
• Malaria	1	3.3
Information provided on infection modalities (*n* = 30)		
• Yes	24	80
• No	6	20
Information provided on healthcare services (*n* = 30)		
• Yes	23	76.7
• No	7	23.3
Status of the illness^a^ (*n* = 30)		
• Cured	29	96.7
• Still ill	1	3.3
Number of options pursued (different types) (*n* = 30 patients)		
• One option	8	26.7
• Two options	18	60
• Three options	4	13.3
Types of options pursued (*n* = 58 occurrences)		
• CSPS	30	53.6
• Self-medication	18	32.1
• District hospital	1	1.8
• National hospital	1	1.8
• Nursing practice	1	1.8
• Clinic	3	5.4
• Tradi-practitioner	2	3.6

^a^ Self-reported 30 days after diagnosis

Total spending for a dengue episode (all options combined) ranged from 1 300 to 67 300 FCFA (1US$ = 477 FCFA), with an average of 13 771 FCFA. Drugs accounted for the largest portion of this total expense, costing on average 5 163 FCFA inside and 5 398 FCFA outside the healthcare center consulted. Those in the lowest income tercile spent on average 8 120 FCFA over one episode (range 2 100–15 150 FCFA), while those in the middle and highest income terciles spent 17 847 FCFA (range 1 300–67 300) and 15 347 FCFA (range 4 050–51 000), respectively. Of the 30 patients, 66.7 % had purchased their prescribed drugs from the healthcare center’s essential generic drugs depot, 83.3 % from a private pharmacy, and 10 % from a travelling salesman. These 30 patients drew upon a variety of sources to pay for their care: salary (50 %), savings (23.3 %), sale of provisions (3.3 %), help from family and friends (20 %), and tontine (i.e., a rotating savings and credit association, 3.3 %).

### Mobility

Mobility, defined as the number of places visited by the patient that were not the patient’s residence, was on average 4.16 for all cases (*n* = 33), of which the majority were within the residence sector (2.8 places visited, vs. 1.75 places visited outside the residence sector). Of the 33 cases of positive RDTs, 11 (33.3 %) reported having travelled outside the capital region, two of whom went outside the country. Eight cases (24.2 %) had gone outside their residence sector and nine cases (27.3 %) had circulated within their sector in the past 12 h.

### Entomological results

**Fig. 4:**
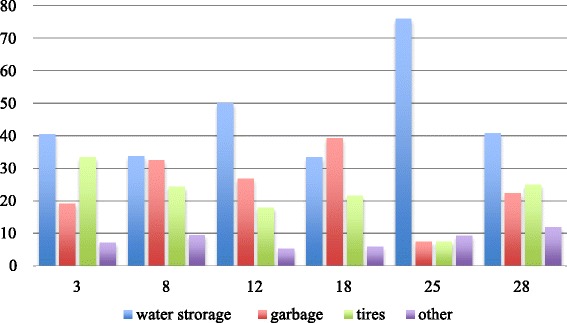
Distribution (%) of types of *Aedes aegypti* breeding sites by sector

In each CSPS’ neighborhood, we surveyed about 20 yards, for a total of 110 randomly chosen yards. The 30 subjects with positive RDT results who were followed up were distributed in 28 households; their yards were also examined, for a total of 138 yards surveyed. In those 138 yards, 356 potential breeding sites were identified (average 2.6 per yard). There were potential *Aedes* breeding sites in the yards of 71.4 % of the localized subjects (20/28). Of the total sites, only one-third were in water (108/356). Four sites that contained water storage (two in sector 18, one in sector 25, and one in sector 12) were found to have *Aedes* larvae (4/108, or 3.7 %). No positive breeding site was found in the patients’ yards. The potential breeding sites encountered most often were water storage containers (terracotta containers or *canaris*, cement cisterns, barrels, or buckets) (45.2 %), garbage left in yards, such as food tins (24.7 %), and tires (21.6 %). Their distribution differed by sector, suggesting heterogeneous behaviors and differences in exposure to *Aedes* proliferation depending on area of residence (Fig. 4). Only *Aedes aegypti* was identified from the larvae collected and reared to adulthood.

However, the risk of *Aedes* proliferation in water storage containers was limited by the fact that 21 % of the households did not store water. Among the households that stored water, 38.8 % used closed containers, 24.1 % did not cover their water containers, and 16.1 % covered them only partially, exposing them to potential *Aedes* colonization. These risky practices were primarily encountered in sector 25 (Somgande: 58 %), followed by sectors 3 (43 %), 8 (41 %), 12 (41 %), 28 (33 %) and 18 (29 %).

**Fig. 5:**
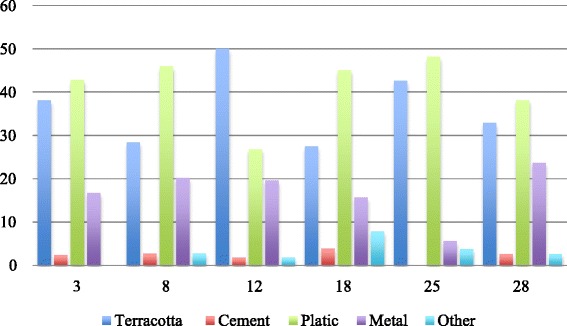
Distribution (%) of the materials involved in *Aedes aegypti* breeding sites by sector

The materials involved in these breeding sites were primarily plastic (41 %) and terracotta (36.2 %), with some variations by sector (Fig. 5).

Table 6 presents the results obtained regarding the adult mosquitoes captured. Most of the captured specimens consisted of *Culex* (97.9 %). With the exception of sector 3, where another species, *Aedes vexans*, was found, only the species *Aedes aegypti* was identified.

**Table 6:** Mosquito genus captured in Ouagadougou by sector

Sectors	No. of *Aedes*(%)	No. of *Anopheles* (%)	No. of *Culex* (%)	Total
CSPS 3 (Dapoya)	3 (0.2)	41 (2.3)	1 768 (97.6)	1 812
CSPS 8 (Gounghin)	13 (2.1)	0 (0.0)	605 (97.9)	618
CSPS 12 (Dapoya)	3 (1.0)	1 (0.3)	310 (98.7)	314
CSPS 18 (Pissy)	5 (1.6)	4 (1.3)	299 (97.1)	308
CSPS 25 (Somgande)	3 (1.8)	0 (0.0)	160 (98.2)	163
CSPS 28 (Dassasgho)	3 (0.5)	7 (1.1)	648 (98.5)	658
TOTAL	30 (0.8)	53 (1.4)	3 790 (97.9)	3 873

DENV was not found in any of the *Aedes* mosquitoes analyzed using PCR. In the first amplification of the nested PCR, cryptic bands were observed but failed to be confirmed in repeat PCR [36].

## Discussion

In this study we found that, even though a large number of patients had positive malaria rapid diagnostic tests, there was also a proportion of the population with febrile episodes non-attributable to malaria. Moreover, among the febrile patients consulting at the selected healthcare facilities in Ouagadougou, there were dengue cases from which it was possible to identify for the first time in the city the simultaneous presence of three DENV serotypes (DENV2, DENV3, DENV4). Given these findings, the scientific community, and more importantly the health workers, need to consider dengue as one possible cause for febrile illness in Burkina Faso.

We observed positive dengue RDTs in every age group. Although 45 % of the subjects were under 15 years old, the largest proportion of dengue cases was seen in patients over age 15 (*p* = 0.001), which is different from patterns observed in Asia but consistent with previous observations in the region and the country [14, 25]. Despite the absence of dengue case notification by the local surveillance system [37], these findings suggest a current ongoing transmission of dengue in the city. The absence of dengue identification and notification in African countries has been attributed to a lack of awareness in the population and in healthcare practitioners and the limited resources available for its diagnosis, among other causes [16, 18, 25, 38]. However, it is known that dengue is and has been present in the African territory and that the absence of information could also be related to the clinical form of presentation in Africans or persons of African ancestry [38–40].

The role of ethnicity and African ancestry in dengue has been widely discussed, and a protective role for severe forms has been described [39, 41–43]. In our study, no severe dengue cases were identified, but warning signs were present in presumptive, probable, and confirmed dengue cases. This finding could be due to a number of situations, such as the presence of secondary infections [13, 44]. Although it was not possible to properly distinguish between primary and secondary infections, due to the presence of positive IgG results and a previously reported 39 % prevalence of DENV IgG in a subsample of Ouagadougou’s population [23, 25], it is conceivable that dengue has been present in Ouagadougou. Likewise, the fact that the majority of dengue cases were adults with pre-existing medical conditions (DM, HTA, liver or kidney disease, etc.) may also have played a role in the clinical manifestations [41, 43, 44]. The presence of warning signs and of symptomatology in general could also be attributed to the presence of DENV3, a serotype known for its virulence and whose presence has been reported in other studies [21, 25, 44–46]. On the other hand, two potential explanations for the absence of severe cases are: 1) the protective role of ethnicity; and 2) the fact that the survey was limited to basic health centers that do not hospitalize patients but instead refer all patients presenting with serious symptomatology to centers providing a higher level of care or to hospitals.

Although the three DENV serotypes were co-circulating in the city, two different serotypes were observed simultaneously in the CSPSs of sectors 8 (Gounghin) and 25 (Somgande). The fact that the majority of DENV3 positive patients were from Somgande could be due to the presence of DENV3 in that sector. Serotype virulence there would lead to people being more symptomatic and more likely to seek healthcare attention. However, the mobility of people living in sector 25 was quite low. The majority of those infected reported not having travelled within the sector nor outside. The low mobility of populations in these sectors is a factor limiting any spread of disease to the whole city. Several studies, using modeling, have shown human mobility to be the primary explanation for disease dissemination in cities in India and Argentina [47, 48].

Even though this study’s findings regarding health expenses and services use are limited because of our small sample, they nevertheless highlight, once more, the challenges of healthcare accessibility and the financial burden these can generate [49]. There have been numerous studies in Burkina Faso on these issues [50], but again, none have looked specifically at dengue. Such studies have yet to be conducted in Africa [49], with particular attention on equity issues. In Cambodia, for example, studies have revealed the extent to which families become indebted to cover expenses related to dengue episodes [51], and the overall financial burden for society and its economy is enormous [52]. The fact that Burkina Faso’s health system is still based on user fees means there is an important financial barrier to healthcare access [50]. New dengue epidemics could exacerbate these impacts for families, especially for the poorest, as was clearly demonstrated in Cambodia [53].

The entomological collections showed that the majority of potential breeding sites found in the sectors surveyed were made up of water storage containers, in plastic and terracotta. Even though the city has made significant advances in its water supply management since the early 1990s (http://www.wssinfo.org), households continue to store water. This practice presents a risk for *Aedes* proliferation, which appeared to exist primarily in sector 25, where water containers were not routinely covered. This result should be confirmed by entomological surveys of the presence of *Aedes* larvae in these containers. In Cameroun, in contrast to what was seen in the rest of Asia, water storage containers were not preferred breeding sites for *Aedes*, which were instead found primarily in abandoned garbage sites and water deposits after rainfalls [54].

### Methodological limitations

It is important to note that this study was conducted rapidly, without the usual time allotted to preparation, because of the need to provide Ministry of Health authorities with rapid data in the context of a new epidemic in the country [9]. As such, this study was conducted after, or at the end of, a dengue outbreak (i.e., an unusually high reported number of cases) in the country after the usual malaria peak and the rainy season. The window of time available was very brief and there was no possibility of conducting additional serological tests (e.g. DENV IgM/IgG ELISA tests) on all the suspected subjects. The absence of specific and confirmatory information on DENV antibodies, together with the fever duration observed, seriously constrained our ability to identify primary and secondary infections or to rule out false positives or confirm false negatives that might have resulted from the limited sensitivity and specificity of RDTs. Moreover, because healthcare attention was focused on the CSPSs level, it was not possible to have the paraclinical work-ups (complete blood counts, liver function tests, etc.) that would have been useful for a complete clinical profile description and to provide a more accurate description when applying the 2009 WHO classification criteria. Also, certain key symptoms (hepatomegaly, abdominal sensitivity to palpation, signs of respiratory distress, etc.) and other clinical signs (blood pressure, pulse) normally used to determine the severity of dengue cases could not be used because they were not covered by the questionnaire and/or there was excessive missing data (more than 50 %). All symptoms not reported in the CSPS register were considered to be absent, even though it was very probable that some (positive tourniquet test, for example) were rarely sought. Even though the results of the entomological survey were somewhat disappointing, given the small number of mosquitoes collected and the absence of productive larval breeding sites, it is highly likely that this was due to the period (end of rainy season) in which the survey was conducted. Interpretation of entomological information was also limited by the fact that 9.1 % of the households were not surveyed, and we do not know whether these were different from the yards surveyed. Although the survey was restricted to only malaria-negative cases as a way of identifying dengue among the febrile non-malaria cases and to decrease the possibility of dengue false positives among malaria cases in the presence of limited confirmatory resources, we acknowledge the possibility of a resultant selection bias. Notwithstanding all the limitations due to the urgency of the need to support decision-makers, we consider this exploratory study was helpful in providing information on the presence of dengue in Burkina Faso and on the challenges involved in studying such events.

## Conclusion

Dengue continues to be a neglected disease in Africa, but because of its emergence or re-emergence, it is becoming urgent that it be given more serious attention and that the positive lessons learned from the malaria journey be applied. This study contributes new and useful knowledge about the presence of dengue virus in Ouagadougou (Burkina Faso). It should help to direct more careful attention to clinical management and elicit more concern from public healthcare actors, in a context where everyone is calling for increased consideration of dengue control in Africa [13, 18]. In Table 7, we summarize the priorities for public health research and interventions highlighted by this article.

**Table 7:** Priorities for public health research and interventions

Research needs:
• Study the seroprevalence and circulation of serotypes.
• Analyze the presence of malaria–dengue co-infection.
• Analyze the health system’s capacity to introduce dengue diagnostic tools during epidemics.
• Analyze the impacts of human mobility on virus circulation.
• Organize entomological studies on circulation, *Aedes* presence, etc.
• Organize interdisciplinary and interventional studies on vector control.
• Study the equity issues raised by dengue.
Public health interventions:
• Mobilize community interventions for vector control.
• Incorporate dengue into the national surveillance system.
• Organize a system to monitor the presence of *Aedes*.
• Train health professionals in dengue management.
• Inform the population about dengue and the means of controlling it.
• Ensure that malaria RDTs are always available and free of charge in CSPSs and that dengue RDTs are available during significant epidemics.
• Reinforce the capacities of the national laboratories.

## Additional file

Additional file 1: Multilingual abstracts in the six official working languages of the United Nations. (PDF 351 kb)

## Abbreviations

ACT: Artemisinin-based combination therapy
AgNS: Antigen of the DENV non-structural protein type 1
CHW: Community health workers
CIDEIM: Centro Internacional de Entrenamiento e Investigaciones Médicas
CSPS: Centre de santé et de promotion social (primary healthcare center)
DEN: Dengue virus
DM: diabetes mellitus
HTA: Hypertension
IgG: Immunoglobulin G
IgM: Immunoglobulin M
LLIN: Long-lasting insecticidal nets
OR: odds ratio
PCA: Principal component analysis
PCR: Polymerase chain reaction
RDT: Rapid diagnostic test
RNA: Ribonucleic acid
RT-PCR: Reverse transcription polymerase chain reaction
WHO: World Health Organization
