# Supplementary material for: Presence of three dengue serotypes in Ouagadougou (Burkina Faso): research and public health implications
Source: Infect Dis Poverty. 2016 Apr 5;5:23. doi: 10.1186/s40249-016-0120-2 (PMC4820922; doi:10.1186/s40249-016-0120-2)
Supplement: Additional file 1: — Multilingual abstracts in the six official working languages of the United Nations. (PDF 351 kb) [file 40249_2016_120_MOESM1_ESM.pdf]

Translation of the abstract into the five official working languages of the United Nations

وجدو ثلاث أنماط مصليه لحمي الضنك في مدينة واغادوغو (بوركينا فاسو): مقتضيات البحث والصحة العامة

فاليري ريد، إيزابيل أجير، إيمانويل بونيت، مابل كارابالي، كونبوبر روش دابير، فلورنس فورنيت، أنتارو لي، إيفلابير بيرتراند ميدا، بياتريس بارا

ملخص

الخلفية: غالبًا ما تفوق عبء مرض الملاريا الكبير في أفريقيا الأمراض الحموية الأخرى. حدث أول ظهور لوباء حمى الضنك في بوركينا فاسو عام 1925 وأخرها عام 2013. ومع ذلك، لا يزال يعرف القليل جدا عن كيفية انتشار مرض حمى الضنك، وناقلات الانتشار الخاص به، وآثاره المرتبطة بالفقر والعدالة.

الطرق: أجريت دراسة تحليل المقطع المُستعرض الاستكشافي من كانون الأول/ديسمبر 2013 إلى كانون الثاني/يناير 2014. تم اختيار عدد ستة مراكز خاصة بالرعاية الصحية الأولية في مدينة واغادوغو استنادًا إلى وجود الفيروسة المُصفرة التي أبلغ عنها في وقت سابق. تمت دعوة جميع المرضي ممن يعانون من الحمي أو الذين عانوا من الحمي خلال أسبوع سابق للخضوع إلى استشارة والمشاركة في اختبار تشخيص سلبي سريع (RDT).تم الحصول على البيانات الديموغرافية، استخدام خدمات الرعاية الصحية والنفقات، التنقل، الحالة الصحية، وممارسات مكافحة الناقلات/ وذلك باستخدام استبيان. تم الحصول على عينات دم من كل مستحق من خلال وخز الإصبع أثناء الدراسة الاستقصائية عن اختبار التشخيص السلبي السريع (RDT) لحمي الضنك باستخدام جهاز (SD BIOLINE Dengue Duo (NS1Ag and IgG/IgM)®) والحصول على بقع دم لتحليل سلسلة من ردود فعل مقايسة المنتسخة العكسية لتفاعل البوليميراز المتسلسل .(RT-PCR) تم العثور على مواقع تكاثر الزاعجة المحتملة ووصفها، في عينة تم اختيارها عشوائياً على مساحة مُحددة على أولئك المرضى. تم جمع وإحضار اليرقات إلى المُختبر لرصد ظهور البالغ منها وتحديد أنواعها.

النتائج: أثبتت نتائج التحاليل أن نسبة 8.7% (379/33) لديهم نتائج اختبارات إيجابية لوجود وباء حمي الضنك. بعد تصنيف منظمة الصحة العالمية عام 2009، وجدت نسبة محتملة أو مؤكدة تقدر بحوالي 38.3% (379/145) لمرض حمي الضنك، استنادًا إلى الأعراض السريرية أو التجارب المختبرية. كما وجد أن 60 عينة من العينات التى تم اختبارها بواسطة RT-PCR (33 من الاختبارات ايجابية و 27 من العينات الثانوية كانت سلبية)، 15 عينة كانت ايجابية. تمت ملاحظة وجود الأنماط المصلية DENV2 و DENV3 و .DENV4 تكون احتمالات الإصابة بحمى الضنك من 15 إلى 20 سنة والأشخاص الذين تتجاوز أعمارهم 50 عامًا كانت 4.0 (CI 95%: 1.0–15.6) و 7.7 (CI 95% : 1.6-37.1) مرات أعلى، على التوالي، من الأطفال دون سن الخامسة. متوسط الإنفاق الكلي لحلقة حمى الضنك هو 771 13 فرانك أفريقي [1 300 – 67 300 فرانك أفريقي] (1 دولار أمريكي = 478 فرانك أفريقي). تم العثور على 2.6 في المتوسط على مواقع التكاثر. تم العثور على مواقع تكاثر الزاعجة المحتملة بالقرب من 71.4% (28/21) من المرضى، ولكن لم يتم العثور على زاعجات بالغة. كانت المواقع المُحددة الأكثر ملائمة لتكاثر الزاعجات عبارة عن صهاريج لتخزين المياه (45.2 %). أكثر العينات التى تم اختبارها كانت البعوضة (97.9%).

الاستنتاجات: ينبغي أن ينظر المجتمع العلمي، وسلطات الصحة العامة، والعاملين في مجال الصحة إلى مرض حمى الضنك كسبب محتمل لمرض الحمى في بوركينا فاسو.

Translated from English version into Arabic by Alain Alameddine, through

TRANSLATORS
WITHOUT BORDERS

瓦加杜古（布基纳法索）有 3 种登革热血清型：对研究工作和公共卫生的影响

Val éry Ridde, Isabelle Agier, Emmanuel Bonnet, Mabel Carabali, Kounbobr Roch Dabir é, Florence Fournet, Antarou Ly, Ivlab èhir é Bertrand Meda, Beatriz Parra

摘要

引言：非洲严重的疟疾负担弱化了其他发热性疾病的重要性。1925 年，布基纳法索第一次爆发登革热，而

最近一次则在 2013 年。但是，我们对登革热的流行情况、相关媒介繁殖情况及其对贫困和公平性的影响知之甚少。

方法：2013 年 12 月至 2014 年 1 月进行了一项探索性横断面调查。选取瓦加杜古曾有黄病毒报道的 6 家初级卫生保健中心。所有因发热就诊或在过去一周内出现发热并经疟疾快速诊断测试（RDT）呈阴性的患者均邀请参与此项调查。采用调查问卷收集社会人口学数据、卫生保健使用和支出、流动性、健康相关状况和媒介控制实践等。通过指尖采集每一名符合纳入的对象的血样，并进行登革热 RDT（试剂盒：SD BIOLINE Dengue Duo，NS1 抗原，IgG/IgM 法）和逆转录 PCR（RT-PCR）检测。随机抽取部分患者家庭庭院，调查潜在的伊蚊孳生地。收集蚊幼虫并带回实验室观察成蚊羽化情况，并鉴别蚊种。

结果：379 份血样中，登革热 RDT 阳性 33 例（8.7%）。基于临床症状或实验室检测，按照 WHO 2009 年的分类，145 例（38.3%）符合可疑、可能及确诊登革热病例。在 60 份经 RT-PCR 检测的样本中（其中 33 份 RDT 阳性、27 份 RDT 阴性），15 份阳性。观察到的血清型有 DENV2，DENV3 和 DENV4。15～20 岁组和 50 岁以上组人群感染登革热的比例分别较 5 岁以下儿童高 4 倍（95%置信区间：1.0～15.6）和 7.7 倍（95%置信区间：1.6～37.1）。平均每发生 1 次登革热平均花费为 13 771 西非法郎（区间为 1 300～67 300，1 美元=478 西非法郎）。每个庭院平均发现 2.6 个孳生点。28 个患者中，21 个（71.4%）家庭庭院中发现潜在的伊蚊孳生点，但是未发现伊蚊成蚊。储水容器是最主要的潜在孳生点（45.2%）。庭院中收集到的大部分蚊虫为库蚊（97.9%）。

结论：科研机构、公共卫生部门及卫生工作者应当考虑到在布基纳法索登革热是发热性疾病的可能病因之一。

Translated from English version into Chinese by Qian Men-bao, edited by Yang Pin, through

CHINESE CENTER FOR DISEASE CONTROL AND PREVENTION
NATIONAL INSTITUTE OF PARASITIC DISEASES

Présence de trois sérotypes de la dengue à Ouagadougou (Burkina Faso) : implications pour la recherche et la santé publique

Valéry Ridde, Isabelle Agier, Emmanuel Bonnet, Mabel Carabali, Kounbobr Roch Dabiré, Florence Fournet, Antarou Ly, Ivlabèhiré Bertrand Meda, Beatriz Parra

Résumé

Contexte : Par le poids important qu’il fait peser sur l’Afrique, le paludisme éclipse souvent d’autres maladies fébriles. La première épidémie de dengue au Burkina Faso s’est produite en 1925 et la plus récente en 2013, et pourtant on connaît très mal la prévalence de la maladie, la prolifération de son vecteur et son impact sur la pauvreté et l’équité.

Méthodes : Une étude exploratoire transversale a été réalisée de décembre 2013 à janvier 2014. Six centres de soins primaires de Ouagadougou ont été sélectionnés en fonction de la présence rapportée du flavivirus. Tous les patients venus fébriles en consultation ou ayant eu de la fièvre au cours de la semaine précédente et pour lesquels le test de diagnostic rapide (TDR) du paludisme était négatif ont été invités à participer à l’étude. Les données sociodémographiques, l’utilisation des services de santé et les dépenses de santé, la mobilité, l’état de santé et les pratiques de lutte contre le vecteur ont été relevés à l’aide d’un questionnaire. Des échantillons de sang de chaque sujet éligible ont été prélevés par ponction sur un doigt au cours de l’étude afin de réaliser un TDR de la dengue avec le test SD BIOLINE Dengue Duo (NS1Ag and IgG/IgM)® et d’obtenir des spots sanguins pour une analyse

par réaction en chaîne à la polymérase en transcription inverse (RT-PCR). Les sites potentiels de ponte d'*Aedes* ont été relevés et décrits dans un échantillon de cours choisies au hasard et dans les cours des patients. Des larves ont été recueillies et rapportées au laboratoire afin de suivre l'émergence des adultes et d'identifier leur espèce.
Résultats : Sur les 379 sujets, 8,7 % (33/379) étaient positifs au TDR de la dengue. Suivant la classification de 2009 de l'OMS, 38,3 % (145/379) avaient une dengue présumée, probable ou confirmée, sur la base soit des symptômes cliniques, soit des tests de laboratoire. Sur 60 échantillons testés par RT-PCR (33 des sujets testés positifs et 27 du sous-échantillon négatif), 15 étaient positifs. Les sérotypes observés étaient DENV2, DENV3 et DENV4. Le risque d'infection par la dengue des personnes âgées de 15 à 20 ans et des plus de 50 ans était 4,0 (IC à 95 % : 1,0-15,6) et 7,7 (IC à 95 % : 1,6-37,1) fois plus élevé, respectivement, que celui des enfants de moins de 5 ans. Les dépenses totales moyennes pour un épisode de dengue s'élevaient à 13 771 FCFA (1300-67 300 FCFA) (1$US = 478 FCFA). En moyenne, nous avons trouvé 2,6 sites de ponte par cour. Nous avons relevé des sites de ponte d'*Aedes* potentiels près de chez 71,4 % (21/28) des patients, mais n'avons pas trouvé de moustiques adultes. Les sites de ponte potentiels les plus fréquemment rencontrés étaient les récipients pour le stockage de l'eau (45,2 %). La plupart des spécimens recueillis dans les cours appartenaient au genre *Culex* (97,9 %).
Conclusions : Il serait utile que la communauté scientifique, les autorités de la santé publique et les professionnels de la santé tiennent compte de la dengue comme cause possible de maladie fébrile au Burkina Faso.

Translated from English version into French by Suzanne Assenat, through

TRANSLATORS
WITHOUT BORDERS

Три серотипа денге в г. Уагадугу (Буркина-Фасо): исследование и потенциальные последствия для системы здравоохранения

Валери Рид, Изабель Ажье, Эммануэль Бонне, Мейбл Карабали, Кунбобр Рох Дабире, Флоранс Фурне, Антару Лай, Ивальбеире Бертран Меда, Беатрис Парра

Реферат

История вопроса: Значительное распространение малярии в Африке часто отвлекало внимание от других лихорадочных заболеваний. Первая эпидемия денге в Буркина-Фасо произошла в 1925 году, а последняя в 2013 году. Однако на настоящий момент очень мало известно о распространенности денге, о пролиферации ее переносчиков и ее воздействии на уровень жизни и равенство населения.
Методы: С декабря 2013 г. по январь 2014 г. было проведено предварительное перекрёстное исследование. На основе данных о наличии флавивируса были отобраны шесть центров первичной медицинской помощи в г. Уагадугу. К участию были приглашены все пациенты, обратившиеся в центр с лихорадкой или страдавшие лихорадкой в течение предыдущей недели, и показавшие отрицательный результат при быстром диагностическом тестировании (RDT) на малярию. С помощью специальной анкеты были собраны социально-демографические данные, данные по обращению в медицинские учреждения и расходам на медицинское обслуживание, данные по подвижности, состоянии здоровья и методам борьбы с переносчиками болезней. У каждого пациента, подходящего для участия в исследовании, была взята кровь пробой из пальца для быстрого диагностического тестирования на денге с использованием набора

реагентов SD BIOLINE Dengue Duo (NS1Ag и IgG/IgM)® и для получения пятен высушенной крови для анализа с полимеразной цепной реакцией с обратной транскрипцией (RT-PCR). В выбранной по случайной схеме выборке подворий и пациентов были определены и описаны потенциальные места размножения комара жёлтолихорадочного (Aedes). Были собраны и доставлены в лабораторию личинки для отслеживания выведения взрослых особей и определения их вида.

Результаты: По итогам быстрого диагностического тестирования 8,7% (33/379) из 379 обследованных лиц показали положительную реакцию на денге. В соответствии с Классификацией ВОЗ 2009 на основании клинических симптомов или данных лабораторных исследований 38,3% (145/379) пациентов был поставлен предположительный, возможный или подтвержденный диагноз денге. Из 60 образцов, на которых был произведен анализ с полимеразной цепной реакцией с обратной транскрипцией (RT-PCR) (33 из тестов с положительным результатом и 27 из субвыборки отрицательных результатов), 15 оказались положительными. Были выявлены серотипы DENV2, DENV3 и DENV4. Вероятность инфицирования денге в возрастных диапазонах от 15 до 20 лет и у лиц старше 50 лет оказалась в 4.0 раза выше соответственно, чем у детей младше пяти лет (CI (доверительный интервал при доверительной вероятности 95%: 1,0–15,6) и 7,7 (CI 95%: 1,6–37,1). Средние общие затраты на один эпизод заболевания денге составили 13771 африканских франков (FCFA) [1 300–67300 FCFA] (1$US = 478 FCFA). В среднем было обнаружено 2,6 мест размножения на одно подворье. Потенциальные места размножения комара жёлтолихорадочного (Aedes) были обнаружены около мест проживания 71,4% (21/28) пациентов, но взрослых особей комара обнаружено не было. Наиболее часто потенциальными местами размножения являлись резервуары для хранения воды (45,2%). Большинство собранных в подворьях экземпляров относятся к виду Culex (97,9%).

Выводы: Научная общественность, органы здравоохранения и медико-санитарные работники должны рассматривать денге как возможную причину лихорадочных заболеваний в Буркина-Фасо.

Translated from English version into Russian by Alena Hrybouskaya, through

Presencia de tres estereotipos de dengue en Ouagadougou (Burkina Faso): investigación y consecuencias sobre la salud pública

Valéry Ridde, Isabelle Agier, Emmanuel Bonnet, Mabel Carabali, Kounbobr Roch Dabiré, Florence Fournet, Antarou Ly, Ivlabèhiré Bertrand Meda, Beatriz Parra

Resumen

Antecedentes: La importante carga de la malaria en África a menudo se ha visto eclipsada por otras enfermedades febriles. La primera epidemia de dengue en Burkina Faso se produjo en el año 1925 y la más reciente en el año 2013. Sin embargo, todavía se conoce poco sobre la prevalencia del dengue, la proliferación de vectores, y su impacto sobre la pobreza y equidad.

Métodos: Se llevó a cabo una encuesta transversal entre diciembre del 2013 y enero del 2014. Se seleccionaron seis centros de atención médica principales en Ouagadougou basándose en reportes previos de presencia de Flavivirus. Se invitaba a participar a todo paciente que consultaba por fiebre o que hubiese tenido fiebre durante

las semanas previas y con negatividad en la prueba rápida de diagnóstico para la malaria. Mediante el uso del cuestionario se capturó información socio-demográfica, de uso de instituciones de salud y gastos, movilidad, condiciones de salud asociadas, y prácticas de control de vectores. Durante la encuesta para la prueba rápida del dengue se obtuvieron muestras de sangre de todos los sujetos elegibles mediante pinchazo en el dedo. Se utilizó SD BIOLINE Dengue Duo (NS1Ag and IgG/IgM)® y para obtener manchas de sangre para el análisis de transcriptasa reversa de la polimerasa (RT-PCR). En una muestra de cultivos seleccionados al azar y de pacientes, se encontraron y describieron posibles sitios de reproducción de Aedes. Se recolectaron larvas y se trajeron al laboratorio para monitorear la emergencia de adultos e identificar la especie.

Resultados: De los 379 sujetos, 8,7% (33/379) fueron positivos para la prueba rápida para el diagnóstico de dengue. Con base en la clasificación del año 2009 de la OMS, en 38,3% (145/379) el dengue se sospechaba, era probable o estaba confirmado, ya sea debido a síntomas clínicos o pruebas de laboratorio. De las 60 muestras evaluadas por RT-PCR (33 de pruebas positivas y 27 de las submuestras negativas), 15 fueron positivas. Los estereotipos observados fueron DENV2, DENV3 y DENV4. Las posibilidades de infección por dengue en personas de 15 a 20 años de edad y en personas mayores de 50 años fueron del 4,0 (IC 95%: 1,0–15,6) y 7,7 (IC 95% : 1,6–37,1) veces más altas, respectivamente, que en los niños menores de 5 años. El gasto total promedio por episodio de dengue fue de 13 771 FCFA [1 300–67 300 FCFA] (1$US = 478 FCFA). En promedio, se encontraron 2,6 sitios de reproducción por cultivo. Se encontraron posibles sitios de reproducción de Aedes en casi 71,4% (21/28) de los pacientes, pero no se encontraron Aedes adultos. Los posibles sitios de reproducción más frecuentemente identificados se encontraban en contenedores para el almacenamiento de agua (45,2%). Los especímenes que más se recolectaron en los cultivos (97,9%) fueron del género Culex.

Conclusiones: La comunidad científica, autoridades de salud pública y los trabajadores de la salud deberían considerar el dengue como posible causa de enfermedad febril en Burkina Faso.

Translated from English version into Spanish by Maria Alejandra Aguada, through

TRANSLATORS
WITHOUT BORDERS
